# Substance P Induces HO-1 Expression in RAW 264.7 Cells Promoting Switch towards M2-Like Macrophages

**DOI:** 10.1371/journal.pone.0167420

**Published:** 2016-12-01

**Authors:** Giovanna Montana, Nadia Lampiasi

**Affiliations:** Istituto di Biomedicina e Immunologia Molecolare “Alberto Monroy”, Consiglio Nazionale delle Ricerche, Via Ugo La Malfa, Palermo, Italy; National Institutes of Health, UNITED STATES

## Abstract

Substance P (SP) is a neuropeptide that mediates many physiological as well as inflammatory responses. Recently, SP has been implicated in the resolution of inflammation through induction of M2 macrophages phenotype. The shift between M1-like and M2-like, allowing the resolution of inflammatory processes, also takes place by means of hemeoxygenase-1 (HO-1). HO-1 is induced in response to oxidative stress and inflammatory stimuli and modulates the immune response through macrophages polarisation. SP induces HO-1 expression in human periodontal ligament (PDL), the latter potentially plays a role in cytoprotection. We demonstrated that SP promotes M2-like phenotype from resting as well as from M1 macrophages. Indeed, SP triggers the production of interleukine-10 (IL-10), interleukine-4 (IL-4) and arginase-1 (Arg1) without nitric oxide (NO) generation. In addition, SP increases HO-1 expression in a dose- and time-dependent manner. Here we report that SP, without affecting cell viability, significantly reduces the production of pro-inflammatory cytokines and enzymes, such as tumor necrosis factor-alpha (TNF-α), interleukine-6 (IL-6), inducible nitric oxide synthase (iNOS) and cyclooxygenase-2 (COX-2), and ameliorates migration and phagocytic properties in LPS-stimulated RAW 264.7 cells. M2-like conversion required retention of NF-κB p65 into the cytoplasm and HO-1 induced expression. Silencing of the HO-1 mRNA expression reversed the induction of pro-inflammatory cytokines in RAW 264.7 stimulated by LPS and down-regulated anti-inflammatory hallmarks of M2 phenotype. In conclusion, our data show that SP treatment might be associated with anti-inflammatory effects in LPS-stimulated RAW 264.7 cells by suppressing NF-κB activation and inducing HO-1 expression.

## Introduction

Substance P (SP) is a neuropeptide mainly produced by primary sensory neurons and stored in peripheral sensory neurons from where it is released after pain stimuli. SP binds to its receptor, the neurokinin-1 receptor (NK-1R), member of the tachykinin subfamily of G protein-coupled receptors, inducing the production of pro-inflammatory cytokines such as IL-6 and TNF-α, which in turn induce the production of NO and ROS, increasing the phagocytosis and antigen presentation in the immune response [1]. The engagement of NK-1R activates members of the MAPK family, such as the extracellular signal-regulated kinases 1/2 (ERK1/ 2), c-jun N-terminal kinases (JNKs) and p38 mitogen-activated protein kinases (p38 MAPK) [1]. These pathways in turn induce the activation and translocation into the nuclei of the transcription factor NF-κB that is the master regulator of inflammation. Indeed, SP has been involved in many inflammatory diseases in which macrophages and mast cells are involved. However, it plays a role in tissue repair after traumatic inflammatory injuries [2] and very recently, SP has been implicated in the resolution of inflammation [3]. Evidence of a role for SP in tissue repair derived from studies which demonstrate its proliferative effect on a variety of cells [4, 5]. Moreover, SP promotes the healing of inflamed colonic epithelium [6] and possess anti-apoptotic effects on many cell types [7]. In fact, SP can act as a mitogen for smooth muscle cells, fibroblasts, endothelial cells and synoviocytes [8–11] and possible plays a role in angiogenesis [12].

Macrophages are cells with great plasticity and versatility depending on the microenvironment signals. In the tissues, macrophages can undergo “classical” activation known as M1 when exposed to toll like receptor (TLR) ligands and interferon-γ (IFN-γ) or “alternative” M2 activation when exposed to IL-4/interleukine-13 (IL-13). However, there is a spectrum of the possible macrophage phenotypes of which M1 and M2 represent the extremes [13]. M1 phenotypes produce pro-inflammatory cytokines such as IL-6 and TNF-α and generate NO and reactive species of oxygen (ROS), whereas M2 phenotypes produce anti-inflammatory cytokines such as IL-10 and IL-4, Arg1 and scavenger molecules. The shift between M1-like and M2-like that allows the resolution of inflammatory processes also takes place by means of HO-1 [14].

HO-1 is a microsomal enzyme that catabolizes heme to obtain biliverdin and carbon monoxide (CO) acting as anti-oxidants. HO-1 is induced in response to oxidative stress and inflammatory stimuli, playing an important role in the suppression of inflammatory reaction and insulin resistance [15]. Indeed, it has been regarded as an adaptive cellular response against NF-κB-mediated inflammation [16] and oxidative cytotoxic conditions, such as excessive production of ROS or TNF-induced apoptosis [17]. HO-1 also exhibited therapeutic benefits in several mouse disease models [18, 19]. Therefore, induction of HO-1 has been thought to produce protective effects against a variety of cellular stresses [20]. The expression of HO-1 is regulated mainly at the transcriptional level [21], although a mechanism of HO-1 degradation through the endoplasmic reticulum-associated degradation pathway has been reported [22]. The promoter sequences of HO-1 contain two enhancer regions (E1 and E2) providing binding motifs for a variety of transcription factors, such as activator protein (AP)-1, cAMP-responsive element binding protein (CREB), NF-κB or Nuclear factor (erythroid-derived 2)-like 2 (Nrf2) [23]. Among these transcription factors, Nrf2, which is the master regulator of the antioxidant stress response, controls many aspects of cell homoeostasis in response to oxidative and toxic insults. In particular, Nrf2 mediates basal and induced transcription of phase II antioxidant proteins, which are responsible for the clearance of NO and ROS, providing protection against the accumulation of toxic metabolites [24].

In order to assess the efficacy of SP as modulator of the inflammatory responses, we evaluated the polarisation towards phenotype M2-like of LPS-stimulated *in vitro* RAW 264.7 cells. Here, we demonstrate that Substance P reduces the levels of inflammatory cytokines and enzymes such as TNF-α and IL-6, iNOS and COX-2 induction and NO production LPS-induced and enhances macrophage switching toward the M2-like phenotype HO-1-mediated. The protective effect of SP on LPS-induced inflammation and the macrophages switching towards M2-like phenotype were confirmed by HO-1 silencing.

## Materials and Methods

### Reagents

Foetal bovine serum (FBS), Dulbecco’s Modified Eagle Medium (DMEM), penicillin and streptomycin (10,000 U/ml) were purchased from GIBCO (Grand Island, NY). LPS from *E*. *coli* serotype 055:B5, Neutral Red, Substance P, Nitrate/Nitrite assay kit colorimetric were purchased from Sigma (St. Louis, MO). DHMEQ was a gift from prof. Umezawa K. TRIZOL reagent, SuperScript Vilo, and Taq DNA Polymerase were obtained from Invitrogen (Carlsbad, CA). RNAiMax transfection reagent was purchased from Invitrogen (Carlsbad, CA). For Western blot analysis, NF-κB p65 (1:1000), lamin B1 (1:1000), Arg1 (1:500) and HO-1 (1:1000) were purchased from Santa Cruz Biotechnology (Santa Cruz, CA, USA). RIPA lysis buffer was purchased from Cell Signaling (Technologies Inc. Beverly, MA, USA). Densitometry analysis was conducted using the Odyssey Infrared Imaging System (Li-COR Bioscience). Incubation with polyclonal mouse β-actin antibody (1:5000) (Sigma Aldrich Srl, Milan, Italy) was performed for comparative control.

### Cell culture and treatment

The murine macrophage-like cell line RAW 264.7 (ATCC) was cultured and treated in DMEM (high glucose 4.5 g/L) supplemented with 10% (v/v) foetal calf serum and antibiotics (100 U/ml penicillin and 100 μg/ml streptomycin) at 37°C in an atmosphere of 5% CO_2_.

### Determination of phagocytosis by neutral red uptake

RAW 264.7 macrophages at 2 X10^5^ cells were seeded onto 8-well chamber slide and allowed to adhere. After 24 hours cells are treated with or without LPS (100 ng/ml) and SP (10 μM/ml) or untreated (Ctrl) for further 24 hours. After incubation, the culture medium was discarded and 200 μl of Neutral Red solution (40 μg/ml in PBS and filtered) was added and cells cultured for another 1 hour. Then the solution was discarded each well was washed three times with Hank’s solution. Lysing solution (200 μl, 0.1 M acetic acid; alcohol 1:1) was added to each well and the plate was incubated 1 hour at 37°C. The absorbance at 492 nm was measured and Hank’s solution served as blank. Experiments were conducted in triplicate and repeated two times. The morphological changes of the cells were observed and photographed at microscopy (20 X magnification). The experiments were repeated two times.

### Nitrate/Nitrite determination

For Nitrate/Nitrite determination, RAW 246.7 cells were seeded in a 24-well plates at a density of 2 × 10^5^ cells/well and grown for 24 hours for adherence. The cells were treated with or without SP (10 μM/ml) and stimulated with LPS (100 ng/ml) for 24 hours. After incubation, the amount of Nitrite in the supernatant was estimated from the accumulation of the stable NO metabolite nitrite. The nitrite concentration in the culture medium was measured as an indicator of NO production according to the Griess reaction by using a kit from Sigma. Briefly, 100 μl of cell culture supernatant was reacted with 100 μl of Griess reagent (1:1 mixture of 0.1% *N*-(1-naphthyl) ethylene-diamine dihydrochloride in water and 1% sulphanylamide in 5% phosphoric acid) in a 96-well plate, and absorbance at 540 nm was recorded using the ELISA reader. The concentration of nitrite in the sample was determined from a sodium nitrite (NaNO_2_) standard curve. The experiments were conducted in triplicate and repeated two times.

### Cell viability assay

Cell viability was determined by use of a trypan blue exclusion assay. Cell (5 X 10^5^) were grown in 24 well plates in complete medium and after 24 hours were treated with or without SP at different concentrations. Cells were scraped coloured with trypan blue and then counted. Experiments were conducted in duplicate and repeated two times.

### RT-qPCR

RAW 264.7 cells were cultured (1 X 10^6^ cells/well) in a 6-well plate overnight. Cells were treated with 100 ng/ml LPS or without (negative control) in the presence or absence of 10 μM SP in DMEM supplemented with 10% bovine serum for 24 hours. Cells stimulated with 100 ng/ml LPS for 24 hours served as a positive control. After 24 hours of stimulation, the cells were detached from the wells and washed once with PBS. Total RNA was isolated using Trizol (Invitrogen Inc.) according to the manufacturer's instructions. The quality and quantity of the RNA samples were determined. Total RNA (3 μg) was converted to cDNA using the SuperScript Vilo (Invitrogen Inc.). QPCR was then performed in triplicate on each cDNA sample for each gene using primer by Qiagen: QT00106169 (IL-10), QT00104006 (TNF-α), QT02418311 (IL-4), QT00134288 (Arg1), QT00098875 (IL-6), QT00159915 (HO-1), PPM0364TE (COX-2), QT00149415 (RelA), QT00100275 (iNOS), QT00103334 (Tacr1), QT01136772 (Actb). The threshold cycle (*CT*) values were calculated against the housekeeping gene *Actb*. For reporting of results, all data were normalised to *Actb*, which was assigned an arbitrary expression level of 10,000, and relative gene expression values were calculated by the following formula: relative expression 10,000/2 *CT*, where *CT* (gene *CT/Actb CT*). Melt curve analysis was conducted to verify the purity and size of the resultant PCR products. At least three distinct biological samples were examined for each gene and treatment (each performed in triplicate).

### Western blot analysis

The RAW 264.7 cells (1 X 10^6^ cells) were cultured in 6-well plates and allowed to adhere for 24 hours. After treatment with SP (10 μM) and LPS (100 ng/ml) the cells were washed twice with cold PBS and nuclei and cytoplasmic fractions were separated by using NER cytoplasmic and nuclear protein extraction kit according to the manufacturer's instructions (Thermoscientif, Pierce). Whole cell lysates were obtained using RIPA buffer (Cell Signaling Inc. Beverly, MA, USA). The protein concentration of cell lysates was determined by the Bradford method. An equivalent amount of protein (30 μg) from whole or nuclear and cytoplasm fractions, respectively, was separated on 10% SDS–polyacrylamide gels by electrophoresis and transferred to a nitrocellulose membrane (Millipore Temecula, CA, USA). The membranes were subsequently incubated for 1 hour at room temperature with 3% BSA in TBS buffer (0.1% v/v) to block non-specific binding and incubated with an appropriate primary antibody in 1% BSA in TBST (tween 0.01% v/v). The secondary antibodies Alexa Fluor 680 goat anti-rabbit (1:2000) and Alexa Fluor 800 rabbit anti-mouse (1:5000), (Molecular Probes, Life Technologies, Carlsbad, CA, USA) were incubated for 1 hour at room temperature. Proteins were visualised using an Odyssey Infrared Imaging System (LI-COR) according to the manufacturer’s instructions. Densitometry analyses were conducted using the Quantity One software (Bio-Rad).

### Small interfering RNA (siRNA) transfection

Cells were transfected with HO-1 siRNA, and Non-Correlated (NC) siRNA (Qiagen, Milan, Italy) as previously reported [25] In brief, cells (2 X 10^5^) were seeded onto 60-mm dishes in medium without antibiotics; 24 h later, the transfection of siRNAs was carried out with Lipofectamine RNAiMAX (Invitrogen). All transfections were carried out with 20 μM duplex siRNA in medium without FBS and antibiotics. After 24 h, cells were split into 6-well plates to perform further analysis. After 24 h (48 h after transfection), cells were untreated or treated for 24 hours with SP and/or LPS and mRNA analysis were performed by RT-qPCR. Experiments were repeated two times.

### Migration assay

Migration assays were performed in 6.5 mm diameter transwell plates containing 8 μm pore size filters (Corning, Costar). Briefly, transwell analysis was conducted after cells were harvested and resuspended (2.0 × 10^5^ cells/ml) in serum-free growth medium. 100 μl of cell suspension (control and treated) was added to the upper chamber whereas in the lower chamber 750 μl of growth medium with 10% fetal calf serum was added with or without SP and LPS. The control group was induced by 10% FCS alone. After 24 h incubation at 37°C, the transwells were removed, the cells were fixed with 4% paraformaldehyde, permeabilized with methanol 100%, colored with crystal violet and then the cells on the upper side were scraped off. The number of cells was counted in 5 random fields under 40 × magnification, and the mean was calculated. The experiments were repeated three times.

### Immunofluorescence microscopy

To stain for NF-κB p65, RAW 264.7 cells were fixed with 4% paraformaldehyde in PBS for 15 min, permeabilized with 0.5% Triton X-100 in PBS for 10 min. The permeabized cells were then blocked with 1% BSA 0.1% Triton X-100 in PBS for 30 min, incubated with primary antibody 1 hour at room temperature (NF-κB p65 1:200, Cell Signaling), and then incubated in the secondary antibody (Alexa Fluor 594 rabbit anti-mouse 1:1000, Invitrogen) and DAPI. Fluorescence images were obtained using a Zeiss Axioskop, Germany. (40 X)

### Statistical analysis

Data are expressed as mean ± S.D. from at least three experiments and statistical analyses were performed by Student's *T* test. *P* < 0.05 was considered to indicate a statistically significant difference.

## Results

### SP Induces HO-1 expression in RAW 264.7 cells

**Fig 1:**
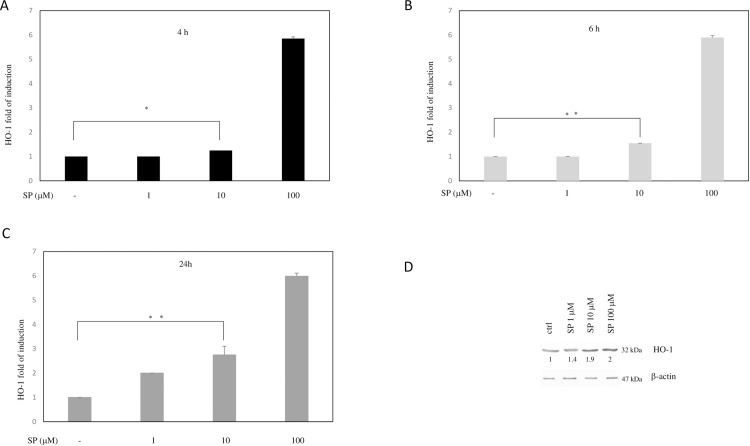
SP induces HO-1 mRNA and protein expression. RAW 264.7 cells were treated with the indicated concentrations of SP for (A) 4, (B) 6 and (C) 24 hours and mRNA expression levels were assessed by RT-qPCR. (B). The results shown are the means ± SD of two experiments (each of which was performed in triplicate). *p<0.05 and **p<0.01 *versus* each agent alone. (D) Cells were treated with SP (μM) for 24 hours. The induction of HO-1 protein expression (32 kDa) was analysed by western blotting. The numbers represent the fold of HO-1 difference with untreated control samples (Ctrl) arbitrarily set at 1.0. The data represent two independent representative experiments.

We first investigated whether the neuropeptide SP could stimulate the production of HO-1 in RAW 264.7 macrophages. Cells were challenged with different SP concentrations (1, 10, 100 μM) and different time of exposure (4, 6, 24 h) and the levels of HO-1 mRNA were quantified by RT-qPCR. Fig 1A, 1B and 1C show a dose-dependent curve response for HO-1 mRNA levels reaching the maximum after a challenge with 100 μM SP. Ten μM of SP incubated for 4 hours caused a significant (p < 0.05) induction of HO-1 mRNA. NK-1R mRNA expression was detected in cells under baseline conditions (data not shown). Next, we evaluated the protein production using the selected doses of SP and exposing macrophages for 24 hours. As expected, SP induced HO-1 protein expression in a dose-dependent manner (Fig 1D). The effect of SP on cell viability was determined by using trypan blue exclusion assay. Neither 10 μM SP nor 100 μM SP had any significant effect on cell viability after 24 hours of incubation (data not shown).

### SP inhibited LPS-induced inflammatory genes expression

**Fig 2:**
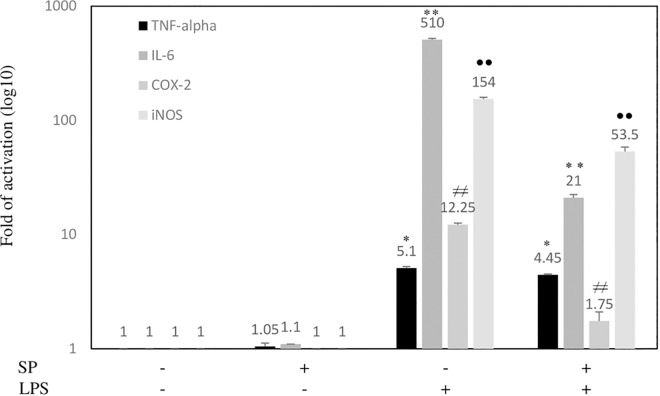
SP decreases TNF-α, IL-6 and COX-2 production in LPS-stimulated cells. RAW 264.7 cells were treated with or without SP (10 μM) followed by treatment with LPS (100 ng/ml) for 24 hours, and the expression of IL6, TNF-α, iNOS,and COX-2 mRNAs was analysed by qPCR. The results shown are the means ± SD of two experiments, each of which was performed in triplicate. * p<0.05 and ** ^## ●●^ p<0.01 *versus* each agent alone.

SP had anti-inflammatory function by increasing IL-10 and decreasing TNF-α *in vivo* [26]. Therefore, we investigated the effects of SP on the pro-inflammatory cytokines expression LPS-induced in RAW 264.7 cells. Non-stimulated cells worked as a negative control, while LPS-stimulated cells worked as a positive control. As shown in Fig 2, the treatment for 24 hours with SP (10 μM) significantly (p < 0.05) reduced the LPS-induced TNF-α, IL-6, iNOS and COX-2 genes. Importantly, addition of SP (10 μM) to RAW 264.7 cells did not induce stimulation. These results suggest an anti-inflammatory effect of SP on the RAW 264.7 treated cells.

### SP induced M2-like macrophages phenotype

**Fig 3:**
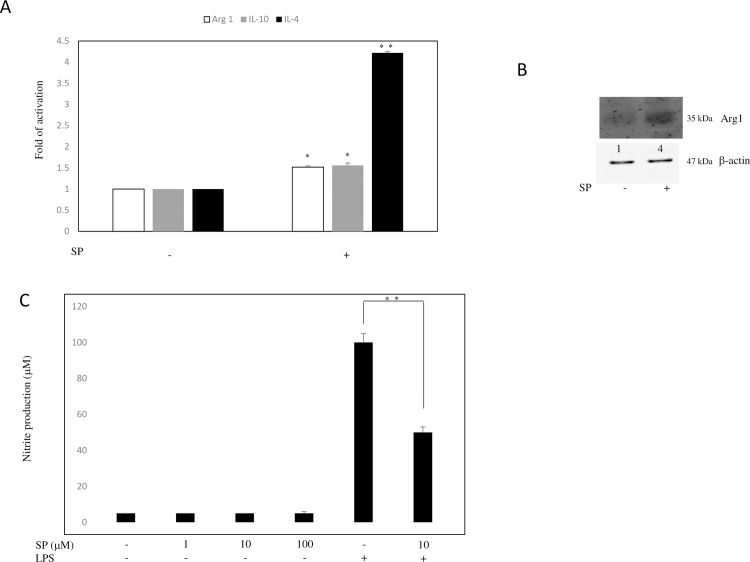
SP induces M2-like phenotype. (A) RAW 264.7 cells were treated with SP (10 μM) and Arg1, IL-10 and IL-4 mRNA expression was evaluated with RT-qPCR. Data are the means ± SD of two experiments (each of which was performed in triplicate). *p<0.05 and **p<0.01 *versus* control. (B) RAW 264.7 cells were treated with SP (10 μM) for 24 hours and Arg1 (35 kDa) protein levels were determined by western blotting. Representative immunoblots of results obtained from three independent experiments are shown. (C) RAW 264.7 cells were treated with different doses of SP or stimulated with LPS 100 ng/ml for 24 hours. NO in the culture medium was measured as described in the methodology section. Data are expressed as nitric oxide concentration (μM) and are the means ± SD of three separated experiments, each of which was performed in triplicate. *p<0.05 versus untreated.

Previous reports indicate that SP induces M2-like macrophages circulating after spinal cord injury [27]. Arg1 and IL-10 are hallmarks of alternatively activated macrophages (M2-like), whereas IL-4 is a potent cytokine that promotes the differentiation of M2 macrophages. To investigate whether SP could modulate the M2-like phenotype, we analysed the Arg1, IL-10 and IL-4 genes expression. As shown in Fig 3A, SP 10 μM induces significant (p < 0.05) Arg1, IL-10 and (p <0.01) IL-4 mRNAs expression. Consistently, we observed an increase of Arginase 1 protein level in RAW 264.7 cells treated with SP (Fig 3B).

M1 macrophages express the enzyme inducible nitric oxide synthase (iNOS), which metabolises arginine to NO and citrulline. Macrophages activated with LPS showed M1-like phenotype; they produce and release NO into cell medium by iNOS protein expression. We treated RAW 264.7 with LPS (100 ng/ml) for 24 hours and then measured NO production into cell medium. As expected, nitrite production was substantially induced as compared to the basal synthesis of the cells, whereas SP treatment did not induce NO production at any of the concentrations tested. When cells were exposed to SP and LPS a significantly reduction of NO production was observed (Fig 3C). Altogether, these results indicate that SP induces M2-like macrophages.

### Effects of SP on LPS-mediated macrophages phagocytosis

**Fig 4:**
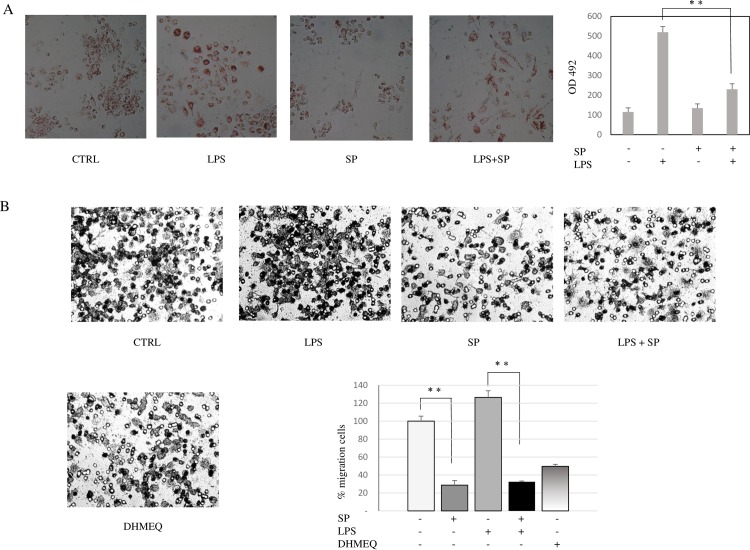
Effects of SP on LPS-mediated macrophages phagocytosis and migration. (A) RAW 264.7 cells were incubated with SP (10 μM) and/or LPS (100 ng/ml) for 24 hours and neutral red phagocytosis was evaluated by adding dye for 1 hour at 492 nm. Data are means ± SD. ** p<0.01 versus untreated. The morphology of the treated cells were visualised by Zeiss microscopy at a 20 x magnification. (B) RAW 264.7 cells were plated on the upper chamber; SP (10 μM) and LPS (100 ng/ml) were used as a chemoattractant in the lower chamber. DHMEQ (2.5 μg/ml) was used as p65 nuclear translocation inhibitor. After 24 hours, quantification of transwells migration was evaluated by counting five random fields per treatment well and averaged across two independent experiments by light microscopy (20 X). The results are presented as migration percent in relation to the total number of untreated cells. Values are means ± SD. ** p<0.01 versus untreated or versus each drugs alone.

High phagocytic capacity is another distinctive function of M1 macrophages [28]. Therefore, we further investigated phagocytic capacity of the macrophages treated with SP in the presence of LPS using the neutral red uptake assay. Morphological results showed that SP stimulated cells were round, stretching out pseudopodia such as M2-like macrophages and showed enhanced phagocytic capacity compared with the control. Instead, LPS treated cells exhibited pseudopodia and elongated fibroblastic-like morphology, showing a different phagocytic maturation as M1-like macrophages (Fig 4A). Association of SP and LPS showed an intermediated appearance between M-1 and M2-like phenotype. The phagocytic function of macrophages was measured by quantitative determination of neutral red uptake. As expected, LPS significantly increased the phagocytic function as compared to control (Fig 4B). Instead, SP does not change the phagocytic function of macrophages. Then, we examined the effects of SP in LPS-induced macrophages. As shown in Fig 4A SP inhibits LPS-induced neutral red phagocytosis. These results indicate that SP induces M2-like macrophages both from resting and activated macrophages.

### Effects of SP on LPS-mediated macrophages migration

Previous studies have demonstrated that LPS mediates inflammatory responses through increasing cell migration to sites of inflammation [29]. To investigate the possible effects of SP on the LPS-induced RAW 264.7 migration, cells were subjected to migration by using the transwell migration assay. RAW 264.7 cells were allowed to migrate for 24 hours in the presence or absence of SP and LPS in the lower chamber. Compared to untreated controls, LPS enhanced, whereas SP significantly (p < 0.01) decreased cells migration. Concurrent use of SP and LPS significantly (p < 0.01) attenuated migration (Fig 4B). To investigate the role of NF-κB transcription factor activation in cells migration, we used dehydroxymethylepoxyquinomicin (DHMEQ), an inhibitor of NF-κB p65 nuclear translocation. As shown in Fig 4B, the migration of cells was significantly (p < 0.05) inhibited in the presence of DHMEQ (2.5 μg/ml). These results suggest that the neuropeptide SP can inhibit migration in activated cells possibly through NF-κB retention into cytoplasm.

### Effect of SP on the activation of NF-κB

**Fig 5:**
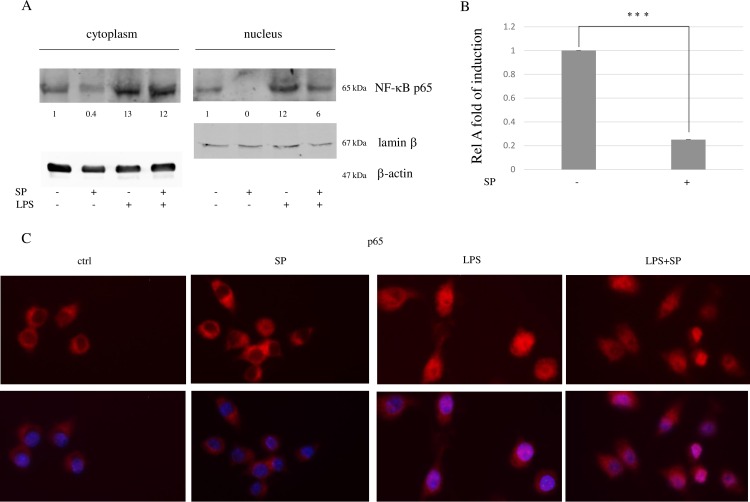
Effects of SP on LPS-induced p65 transcription factor nuclear translocation. (A) RAW 264.7 cells were treated with or without SP (10 μM) and/or with LPS (100 ng/ml) for 4 hours and cytoplasmic and nuclear fractions were separated and analysed by western blotting for NF-κB p65 expression. We used β-actin (47 kDa) and lamin B1 (67 kDa) immunolabelling as loading controls for cytoplasmic and nuclear fractions, respectively. The numbers represent the fold difference of NF-κB p65 with untreated control (Ctrl) arbitrarily set at 1.0. The data shown represent two independent experiments with comparable outcomes. (B) RT-qPCR analysis of p65 (Rel A) mRNA in cells treated or untreated with SP (10 μM) for 4 hours. The data are mean ±SD of two separate experiments, each of which was performed in triplicate. **p < 0.01 versus untreated. (C) Immunofluorescence analysis of RAW 264.7 cells with NF-κB p65 (red) in cells treated with or without SP (10 μM) and LPS (100 ng/ml) for 4 hours. The nuclei of the cells have been counterstained with DAPI (blue). Scale bar 20 μm. Results are shown from two independent experiments with comparable outcomes.

To further define the downstream effectors of the anti-inflammatory effects of SP, we analysed nuclear translocation of NF-κB. RAW 264.7 cells were treated with SP and stimulated with LPS for 4 hours and western blotting analysis was performed. Untreated cells worked as a negative and LPS treated cells as a positive control. As expected, LPS treatment induced an NF-κB increase in the nuclear fraction (Fig 5A). Accordingly, a decrease of the amount of NF-κB p65 was observed in the cytosolic fraction (Fig 5A). Surprisingly, SP alone inhibits p65 expression (Fig 5A and 5B). SP significantly decreased the activation of NF-κB p65 LPS-induced, suggesting that SP exerts its anti-inflammatory effect through p65 cytoplasmic retention. Indeed, strong nuclear NF-κB p65 immunoreactivity was observed in cells exposed to LPS, whereas when cells were exposed to LPS in combination with SP p65 showed cytoplasmic retention (Fig 5C).

### HO-1 induction promotes M2 macrophage polarisation

**Fig 6:**
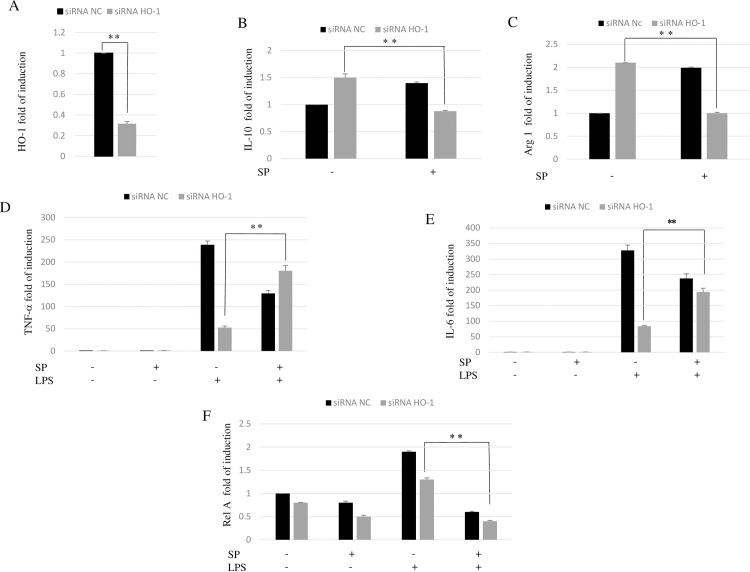
HO-1 induction promotes M2 macrophage polarisation. RAW 264.7 cells were transfected with siRNA HO-1 and siRNA NC (non-correlated). (A) RT-qPCR analysis of HO-1 mRNA in cells transfected 48 hours after transfection. Cells were treated with SP (10 μM) and/or LPS (100 ng/ml) for 24 hours and mRNA expression of selected genes was evaluated by RT-qPCR (B) IL-6 mRNA expression (C) TNF-α mRNA expression (D) Arg1 mRNA expression (E) IL-10 mRNA expression and (F) Rel A mRNA expression. The data are mean ±SD of two separate experiments, each of which was performed in triplicate. **p < 0.01 versus siRNA NC.

HO-1 has potent anti-inflammatory effects on macrophages, promoting the switch between M1 and M2 macrophages [30]. To assess whether HO-1 has a role in promoting the switch between M1-M2-like phenotypes, we transfected RAW 264.7 with the HO-1 siRNA and then treated cells with SP in the presence or absence of LPS for 24 hours. Subsequently, we evaluated the production of pro-inflammatory (TNF-α and IL-6) and anti-inflammatory (IL-10 and Arg1) cytokines with RT-qPCR. As shown in Fig 6, HO-1 silencing significantly (p < 0.05) blocked the upregulation of M2 markers (IL-10 and Arg1) and the downregulation (p < 0.01) of M1 markers (TNF-α and IL-6) transcripts (Fig 6B, 6C and 6D). To further investigate the role of HO-1 as anti-inflammatory factor, we evaluated the expression levels of NF-κB p65 (Rel A) with RT-qPCR. As expected, in consequence of HO-1 silencing, we found a significant (p < 0.01) decrease of the Rel A mRNA expression after LPS and SP/LPS treatment (Fig 6F). Altogether, these results suggest that HO-1 has a role in promoting the switch between M1- and M2-like phenotypes.

## Discussion

Macrophages polarisation between M1 and M2 phenotypes is an important mechanism for the regulation of inflammatory response. In fact, some inflammatory diseases are frequently associated with changes in macrophage activation, with classically activated M1 cells implicated in initiating and sustaining inflammation and M2 or M2-like cells associated with resolution or dampening inflammation. M1 macrophages produce large amounts of pro-inflammatory cytokines such as TNF-α, COX-2 and IL-6, which can generate NO and ROS and express high levels of MHC molecules functioning as killers of pathogens and tumour cells. In contrast, M2 macrophages produce high quantities of IL-10 and Arg1, express scavenger receptors and molecules and exhibit anti-inflammatory and tissue repair functions [31]. However, it is unlikely that macrophages *in vivo* exist in such clear distinct phenotypes. Instead, there probably coexist intermediate activated subsets, because many stimuli are present at the same time in the tissues. Interestingly, polarisation of macrophages may depend on cellular types and stimuli, and the same stimuli can promote pro- or anti-inflammatory macrophages in different circumstances.

The production of pro-inflammatory cytokines and oxidative metabolites are key effectors of M1-like phenotype, while the down-regulation of these inflammatory cytokines, coupled with enhanced production of anti-inflammatory cytokines, are effectors of M2-like phenotype. LPS acting via Toll-like receptor-4 (TLR-4) enhances the expression of a number of inflammatory genes, such as TNF-α, IL-6, iNOS and COX-2, principally through activation of a number of transcription factors including AP-1 and NF-κB [32]. SP is a neuropeptide also found in non-neural cells such as mast cells [33], macrophages, lymphocytes and dendritic cells [34]. It stimulates immune cells to produce inflammatory cytokines, promotes vasodilatation, tissue repair and migration [34]. In the current study, RAW 264.7 cells treated with SP did not exhibit TNF-α, COX-2 and IL-6 expression neither NO production. However, other works have shown that SP can increase pro-inflammatory cytokines [35, 36]. It has been taken into account that in these studies, additional stimuli were present which can alter the response of the cells. In particular, in all works, the stimulation with SP occurred 2 hours after the deprivation of serum. Some transcription factors, such as NF-κB and p38 MAPK, were activated in this situation [37] and could induce pro-inflammatory cytokines. Here, we have characterised an anti-inflammatory role of SP that leads to a shift in M2-like macrophages phenotype, both of resting and activated cells. In fact, we found that RAW 264.7 macrophages in response to SP produce IL-10, IL-4 and Arg1, that are important hallmarks of M2 macrophages. Instead, LPS-stimulated RAW 264.7 macrophages resulted in a significant increase in nitrite levels and an upregulation of iNOS and COX-2 expression. In our study, SP pre-treatment in RAW 264.7 cells LPS-stimulated resulted in a significant down-regulation of COX-2 expression, indicating its potential anti-oxidant effect. In addition, the anti-inflammatory effect of SP was accompanied by suppression of inflammatory mediators such as TNF-α and IL-6 LPS-induced, which could be achieved through retention of NF-κB transcription factor in the cytoplasm. In fact, inhibition of NF-κB p65 activation LPS-induced was achieved through SP treatment indicating an anti-inflammatory effect of SP. Accordingly, other authors found that anti-inflammatory effects on RAW 264.7 of the molecules are exerted through NF-κB suppression [38]. NF-κB is also directly involved in pro-inflammatory responses such as adhesion and migration of leukocytes. It should be noted that LPS could promote leukocyte migration *in vivo* and RAW 264.7 migration *in vitro* [39]. In our study, we found that LPS promotes macrophage migration and that SP treatment inhibits LPS-induced migration. Inhibition of migration is dependent on suppression of NF-κB transcription activity on MMP-9 expression [40]. Herein, we showed that DHMEQ, an inhibitor of NF-κB p65, significantly (p < 0.05) reduced migration in RAW 264.7 cells.

Induction of HO-1 is able to mediate potent anti-inflammatory and other beneficial effects via the release of CO [41–43]. HO-1 expression inhibits the production of inflammatory cytokines and chemokines, such as IL-1β and IL-6 induced by activated macrophages [44, 45]. Indeed, upregulation of HO-1 expression suppresses LPS-induced inflammatory responses [41, 45]. Herein, SP treatment of RAW 264.7 cells significantly increase HO-1 mRNA and protein expression in a dose-dependent manner. Thus, upregulation of HO-1 expression in SP-treated RAW 264.7 cells could inhibit inflammatory response. Indeed, in our experiments, the silencing of HO-1 expression SP-induced reverted the downregulation of inflammatory cytokines in activated RAW 264.7 (TNF-α and IL-6). HO-1 enhances the expression and activity of peroxisome proliferator-activated receptor gamma (PPARγ), and the upregulation of PPARγ may promote the polarisation towards the M2 phenotype. SP enhances PPARγ protein expression in human monocyte/macrophage [46]. The anti-inflammatory potential of PPARγ mainly resides in the ability of its agonists to inhibit monocyte/macrophage activation and expression of inflammatory molecules, i.e. TNF-α, IL-6, iNOS and COX-2 [47, 48]. It should be noted that PPARγ might control the inflammatory response through AP-1 and NF-κB in activated M1 macrophages [49]. Moreover, PPARγ directly controls the expression genes such as Arginase I involved in inducing the M2 macrophage phenotype [50]. In our study, we showed that knockdown of HO-1 blocked the upregulation of M2 markers (IL-10 and Arg1) in SP treated cells. Although the exact mechanisms involved in anti-inflammatory effects of HO-1 have not been fully elucidated, the interference with the transcription factors NF-κB pathway seems to be the most probable. Indeed, in our experiments, HO-1 knockdown decreased NF-κB p65 mRNA expression.

## Conclusions

**Fig 7:**
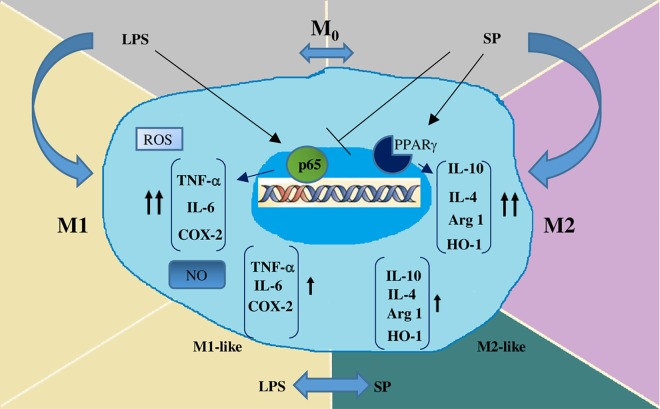
Immunomodulatory effects of Substance P in the M1- to M2-like phenotype conversion of RAW 264.7 cells.

In conclusion, our results suggest that SP exerts its anti-inflammatory effect in LPS-stimulated RAW 264.7 cells by suppressing NF-κB activation and inducing HO-1 expression. To our knowledge, this is the first paper that attempts to identify in macrophages signalling transduction mechanisms used by SP to promote anti-inflammatory effects. As summarised in Fig 7, this study highlights the effect of SP in inducing the M2-like phenotype and its ability to promote M1- to M2-like phenotypical conversion.
